# Smart Textile Based on Fiber Bragg Grating Sensors for Respiratory Monitoring: Design and Preliminary Trials

**DOI:** 10.3390/bios5030602

**Published:** 2015-09-14

**Authors:** Marco Ciocchetti, Carlo Massaroni, Paola Saccomandi, Michele A. Caponero, Andrea Polimadei, Domenico Formica, Emiliano Schena

**Affiliations:** 1 Unit of Measurements and Biomedical Instrumentation, Center for Integrated Research, Università Campus Bio-Medico di Roma, Via Álvaro del Portillo, 21, Rome 00128, Italy; E-Mails: marco.ciocchetti@alcampus.it (M.C.); c.massaroni@unicampus.it (C.M.); p.saccomandi@unicampus.it (P.S.); 2 Photonics Micro- and Nano-structures Laboratory, Research Centre of Frascati, ENEA, Via E. Fermi, 45, Frascati, Rome 00044, Italy; E-Mails: michele.caponero@enea.it (M.A.C.); andrea.polimadei@enea.it (A.P.); 3 Unit of Biomedical Robotics and Biomicrosystems, Center for Integrated Research, Università Campus Bio-Medico di Roma, Via Álvaro del Portillo, 21, Rome 00128, Italy; E-Mail: d.formica@unicampus.it

**Keywords:** smart textile, magnetic resonance-compatible, fiber optic sensors, fiber Bragg grating sensors, optoelectronic plethysmography, respiratory monitoring

## Abstract

Continuous respiratory monitoring is important to assess adequate ventilation. We present a fiber optic-based smart textile for respiratory monitoring able to work during Magnetic Resonance (MR) examinations. The system is based on the conversion of chest wall movements into strain of two fiber Bragg grating (FBG) sensors, placed on the upper thorax (UT). FBGs are glued on the textile by an adhesive silicon rubber. To increase the system sensitivity, the FBGs positioning was led by preliminary experiments performed using an optoelectronic system: FBGs placed on the chest surface experienced the largest strain during breathing. System performances, in terms of respiratory period (T_R_), duration of inspiratory (T_I_) and expiratory (T_E_) phases, as well as left and right UT volumes, were assessed on four healthy volunteers. The comparison of results obtained by the proposed system and an optoelectronic plethysmography highlights the high accuracy in the estimation of T_R_, T_I_, and T_E_: Bland-Altman analysis shows mean of difference values lower than 0.045 s, 0.33 s, and 0.35 s for T_R_, T_I_, and T_E_, respectively. The mean difference of UT volumes between the two systems is about 8.3%. The promising results foster further development of the system to allow routine use during MR examinations.

## 1. Introduction

The continuous respiratory monitoring is essential in different medical fields. For instance it is crucial during anesthesia, for evaluating sleep apnea disorders, for monitoring of infants susceptible to Sudden Infant Death Syndrome (SIDS), and recently has been proposed to evaluate the coordination between nutritive sucking and respiration in preterm infants, to provide standardized and reliable measuring methods in assessing newborns’ neuro-motor status [1,2]. Several commercial devices monitor the efficacy of the respiration by assessing the blood oxygenation, the respiratory rate (*i.e.*, the number of respiratory acts taken within a minute), the duration of inspiratory and expiratory phases, and estimate the tidal volume (*i.e.*, the air volume inhaled or exhaled in a single breath). In most cases these devices cannot be used during procedure of Magnetic Resonance (MR) examinations. Two main reasons motivate the need to monitor respiration (e.g., breathing frequency, respiratory volume exchanges): (i) often, patients undergoing these procedures experience anxiety and panic symptoms. These events can be detected early by respiratory rates, avoiding patient discomfort; (ii) the use of MR is continuously increasing worldwide (for instance more than 20,000 MR scanners are present in Economic Cooperation and Development countries [3]). Therefore, the necessity for devices able to work in MR room (*i.e.*, MR-compatible systems), and providing the estimation of respiratory parameters during MR procedures is growing considerably.

In this arena the development of sensors based on fiber optic technology is gaining large interest in the scientific community and its market is growing worldwide [4]. Fiber optic sensors (FOSs) are immune from electromagnetic interferences, moreover fiber optics are made either of glass or of polymer (polymer optical fiber, POF) allowing to develop MR-compatible sensors, able to measure different chemical, thermal, and mechanical parameters [5]. Among their advantages there are: good static and dynamic properties (*i.e.*, low zero drift and sensitivity drift, good accuracy, good sensitivity, and large bandwidth) and the possibility to develop distributed sensors; moreover POF are able to undergo high strain without damage. Lastly, they are intrinsically safer than electric sensors because they do not require electrical connection to the patient. These advantages foster the use of FOSs in several medical fields, such as orthopedics, respiratory monitoring, and cancer ablation, among others [5,6,7,8]. During last decade, the development of FOS-based smart textiles is gaining increasing popularity, thanks to their small size, the possibility to sense and transmit the signal along the same fiber, and the MR-compatibility [9,10]; moreover solutions based on textile body-monitoring are less expensive than in-patient treatments allowing a reduction of the treatment costs. The growing interest of the scientific community around smart textiles, FOSs and their integration is witnessed by the vast scientific literature and recent reviews, by the large number of international annual conferences focused on these fields, and by the project funding worldwide, such as the project OFSETH [9,10,11]. This interest is also confirmed by the recently published work focusing on the technologies suitable for application in MR procedures [12]. Furthermore, the possibility to monitor physiological parameters during MR procedures motivates the interest around FOS-based smart textiles, because they cannot be replaced by other very interesting solutions, such as patch monitors [13,14].

FOS-based smart textiles are considered a good solution for respiratory monitoring and a potential new market niche in the field of healthcare monitoring [14,15,16]. In fact, they can answer to the increasing demand for both MR-compatible systems and wearable systems for continuous monitoring of respiratory parameters. Several groups of research are focusing their activities on this topic mainly using FOSs based either on macrobending or on fiber Bragg grating (FBG) technology [15,16,17,18,19,20]. Two main solutions are proposed to develop smart textiles: (i) by embedding the FOS within the textile; (ii) an easier solution embeds FOS within a polymeric layer glued on the textile [1,19].

The aim of this work is to assess the feasibility for respiratory monitoring of an MR-compatible smart textile based on FBG technology. The main novelty of the proposed sensing system is that the positioning of the FBGs on the textile is led by an optoelectronic system, OS, (D-Smart™, BTS Bioengineering Corp., Milan, Italy). This solution has been adopted in order to increase the sensitivity of the system by two steps: (i) the OS allowed estimating the chest wall movement during breathing; (ii) FBGs were positioned on the chest site, which experienced the largest strain.

The smart textile was tested on four healthy volunteers and its performances in estimating the respiratory period (T_r_), as well as duration of inspiratory (T_i_) and expiratory (T_e_) phases were assessed by using as reference the measurements recorded by an optoelectronic plethysmography system (*i.e.*, OEP™, BTS Bioengineering Corp., Milan, Italy). The OEP™ consists of the mentioned OS and a dedicated software which allows estimating the respiratory volumes starting from the 3D markers coordinates. Lastly, the correlation between the output of the two FBGs and the left (UT_L_) and right (UT_R_) upper thorax volumes recorded by the OEP was made to investigate the feasibility of the proposed system to estimate these quantities.

## 2. Design and Fabrication of the Smart Textile

The working principle of the smart textile is based on the monitoring of the chest wall deformation during breathing. Among several ways to locally measure the deformation of a textile, we focused on sensors based on FBG technology. We used two commercial FBGs inscribed in a single optical fiber with the aim to assess the deformation of UT_L_ and UT_R_. The length of the two FBGs was 1 cm, and we employed glass fiber optics (SMF 28, Corning^®^, New York, NY, USA).

Basically, a FBG can be considered as a short segment of a fiber optic, which reflects a narrow range of wavelengths and transmits all others. The wavelength of the input light that is back-reflected (λ_B_) is sensitive to temperature and strain; as a consequence, the use of a proper configuration allows estimating strain and temperature or both, by monitoring changes of λ_B_ [22].

The main advantage of using FBG is the linear response to strain; furthermore, the chemical inertness, as well as the small size, flexibility, and MR-compatibility make them particularly attractive for our application [3].

In the design of the proposed smart textile it has been essential to ensure the best placement of the FBGs on the two sides of the UT.

**Figure 1:**
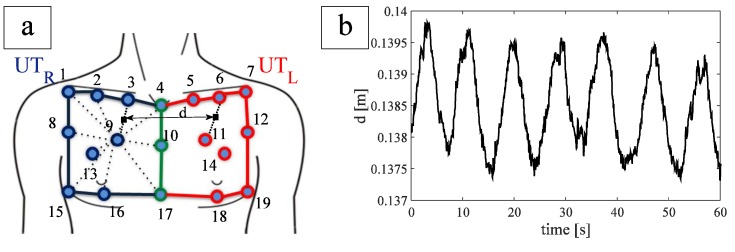
(**a**) FBGs position and distance between the two FBGs. Blue lines and markers identify the upper thorax right compartment, red lines and markers identify the upper thorax left compartment, green lines and markers identify the line which separates the two compartments; (**b**) trend of FBG distance during quiet breathing of a healthy subject.

To optimize the placement of FBGs on the textile, six healthy subjects (mean age 45 ± 4 years, mean chest circumference 97.7 ± 8.8 cm) underwent OS to investigate the UT kinematic. We focused our attention on the UT_L_ and UT_R_ (see Figure 1a). The thoracic motion was recorded by 19 passive photo-reflective markers placed on approximately horizontal rows at the levels of the clavicular line, the manubriosternal joint, and the nipples, as recommended during the analysis of chest wall motion [23].

The inter-marker distance was calculated for each couple of markers identifying one of the two compartments during the whole breathing. It allowed estimating the UT movements and inter-marker distance experienced for each compartment during breathing with the aim to optimize the FBGs positioning on the chest.

The largest variation of inter-marker distance during breathing was found on the line joining markers 9 and 3 (~2.54 mm) and markers 11 and 6 (~3.29 mm), for UT_R_ and UT_L_, respectively. These two variations were recorded on the same patient.

In order to embed the two FBGs within a single optical fiber, we studied the distance d (the virtual midpoint of both segment 3–9 and 6–11, Figure 1a during breathing). The averaged d value, considering all the subjects, were 14.0 ± 2.3 cm. In order to facilitate the wearability of the smart garment, we made an optical fiber with an FBGs inter-distance of 18 cm, by considering a safety factor of ~30%.

Two FBGs were spliced one-sidedly with a pigtail ending and attached to a single fiber optic for angled physical contact (FC/APC)-type connector capable of connection to an interrogation system. FBGs with 10 mm of grating length, strain sensitivity of about 1.2 pm∙µε^−1^, and different nominal λ_B_ (*i.e.*, 1532 nm and 1541 nm) were chosen.

The fiber optic was fixed into a commercial slim fit size-L T-shirt (OXYLANE^©^, 97% polyamid, 3% elastane, Kipsta, Villeneuve-d’Ascq, France) in two sites by an adhesive silicon rubber containing trimethoxy(methyl)silano (3145 RTV MIL-A-46146, DOW CORNING, Michigan, MI, USA) of about 15 cm^2^. Silicon rubber was left cure to a rubbery solid for 72 h, at room temperature.

## 3. Materials and Methods

### 3.1. Experimental Set-Up and Protocol

**Figure 2:**
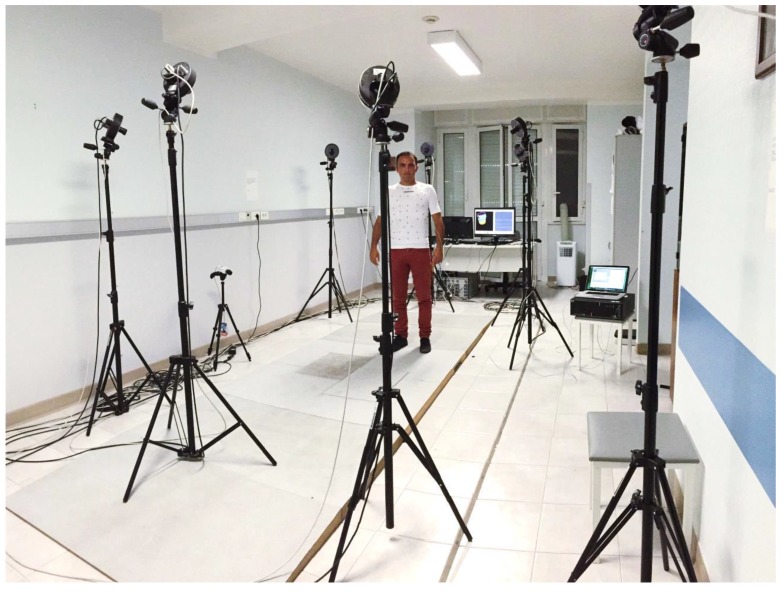
Picture of the experimental set-up.

The performances of the smart textile in terms of respiratory period, inspiratory and expiratory period, as well as UT_L_ and UT_R_ estimation were investigated on four healthy volunteers. The respiratory monitoring of the volunteers was performed during quiet breathing by the smart textile and by the OEP with the 89-markers protocol, as shown in Figure 2. OEP is largely employed for respiratory monitoring and non-invasive assessment of breathing diseases, thanks to its accuracy and precision [24]. OEP is based on typical methods for optical motion analysis and allows evaluating ventilatory volumes through an external measurement of the chest wall surface motion recording the 3D position of passive photo-reflective markers. Accordingly to the literature, 89 IR-reflective passive markers were placed on the trunk of the each subject in selected anatomical reference sites of the upper thorax, the rib cage, and the abdomen by the use of hypoallergenic tape [25].

The subject was placed in the center of a room where eight TV cameras, operating at 60 Hz, were conveniently positioned, Figure 2. OEP determines the three-dimensional coordinates of each marker by means of trademarked software (OEP-Smart™, BTS Bioengineering Corp.), that is able to compute volumes starting from the three-dimensional coordinates of markers. The raw data representing the volumes are exported in an Excel file and then imported in MATLAB^®^ (MathWorks, Inc., Massachussetts, MA, USA) environment.

The output of the two FBGs embedded in the textile was recorded with a sample frequency of 250 Hz by an optical spectrum analyzer (si425 Reading Unit, Micron Optics Inc., Figure 2), exported in an Excel file, and then imported in MATLAB^®^ environment.

Four healthy subjects, with no history of respiratory disorders, have been enrolled (all males, mean age 28 ± 4 year, mean thoracic circumference 98 ± 3 cm). Each subject was forced to do two sessions lasting 1-min of quiet breathing in a standing position, wearing the textile.

### 3.2. Data Analysis

Data obtained by both the textile and the OEP system were elaborated in MATLAB^®^ environment. In particular, from the optical spectrum analyzer, the wavelengths of the two FBGs were recorded and processed off line.

**Figure 3:**
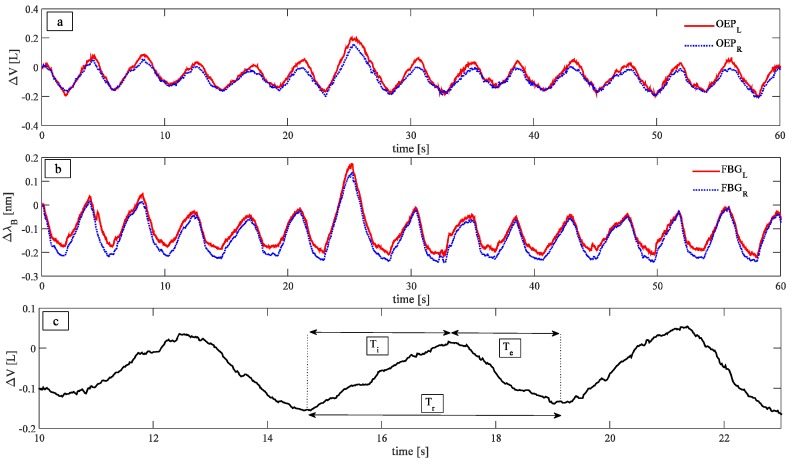
(**a**) Trend of the data provided by the OEP; (**b**) Trend of the data provided by the two FBGs; (**c**) three different parameters investigated: Respiratory periods, calculated as the time interval between two consecutive peaks, inspiratory periods, calculated as the time interval that elapses between a maximum and the previous minimum of the signal, and expiratory periods, calculated as the time interval that elapses between a minimum and the previous maximum.

A typical trend of the data wavelength provided by the two FBGs, as well as the variation of UT_R_ and UT_L_ volumes estimated by OEP are shown in Figure 3a,b.

Signals peaks were selected in order to compare T_r_, T_i,_ and T_e_ estimated by the smart textile and the OEP, as well as to correlate variations of wavelength with changes in the UT volume estimated by the OEP system for both left and right sides. Two methods were used to select the peaks: An automated method, implemented in MATLAB environment, and a second method, based on a manual selection of peaks. The automatic method allows performing the task faster than the manual one; on the other hand, the manual approach may be used to select just a part of the patterns, to exclude specific respiratory periods when it is necessary.

In order to investigate the ability of the system to estimate T_r_, T_i_, and T_e_ phases, the following parameters were calculated, as in Figure 3c: (i) T_r_, as the time interval between two consecutive peaks; (ii) T_i_, as the time interval that elapses between a maximum and the previous minimum of the signal; and (iii) T_e_, as the time interval that elapses between a minimum and the previous maximum. The measurements provided by the OEP were considered as reference, to assess the performances of the smart textile.

Furthermore, a preliminary analysis regarding the ability of the system to estimate UT values was carried out by calculating the correlation between the wavelength changes of the two FBGs and changes of the UT volumes measured by OEP. Both FBGs output and UT volume trend for the same respiratory act were extracted; since these signals were recorded with different sample frequencies, they were normalized with respect to the act duration (0%–100% act). This normalization allowed performing the linear fitting of FBGs wavelength *vs.* the corresponding UT volume. The sensitivity, considered equal to the slope of the best fitting line, was calculated for each act. In order to provide for each patient a single calibration curve the averaged-sensitivity on all the acts was calculated.

Using the mean value of the sensitivity, the measurement error of the system in UT_L_ and UT_R_ estimation, was calculated as follows:

(1) $$e=\frac{\left(peak_{MAXFBG}\left(i+1\right)-peak_{\min FBG}\left(i\right)\right)}{k}-\left(peak_{MAXOEP}\left(i+1\right)-peak_{\min OEP}\left(i\right)\right)$$

where peak_MAXFBG_(*i* + *1*) and peak_MAXOEP_(*i* + *1*) are the *i* + *1*th maximum value of the FBG wavelength and the *i* + *1*th maximum of UT volume, peak_minFBG_(*i*) and peak_minOEP_(*i*) are the *i*th minimum value of the FBG wavelength and the *i*th minimum of the UT volume, *k* is the mean value of the sensitivity. Then, the percentage error was calculated as follows:

(2) $$\%e=\frac{error}{UT\;volume}\cdot 100$$

where the UT volume was calculated as the difference between the *i* + *1*th maximum and the *i*th minimum recorded by OEP.

The measurements performed by the smart textile were compared with the ones made by the OEP by using Bland Altman method [26]. The mean of difference (MOD) and limits of agreement (LOA) values for all the parameters of interest were calculated. MOD can be considered as index of the accuracy of the method under test; LOA = MOD ± 1.96∙std, where std is the standard deviation of the difference between the two methods, representing the range including 95% of differences between the two measurement methods.

## 4. Results

**Figure 4:**
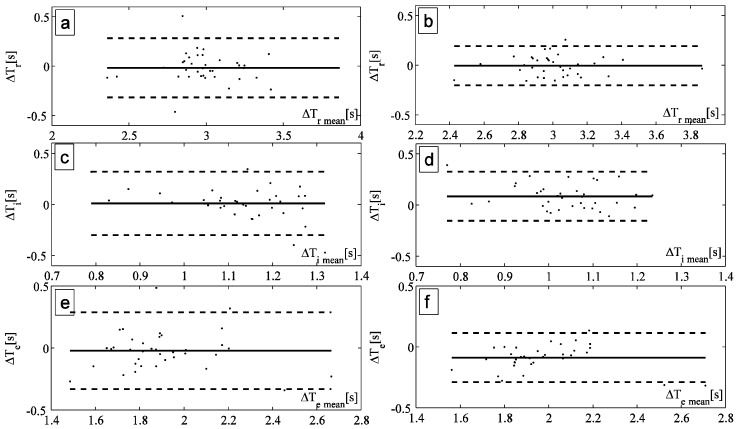
(**a**,**b**) Bland Altman plot comparing the respiratory period measured by OEP and by the smart textiles with the automatic method and the manual one, respectively; (**c**,**d**) Bland Altman plot comparing the inspiratory period measured by OEP and by the smart textiles with the automatic method and the manual one, respectively; (**e**,**f**) Bland Altman plot comparing the expiratory period measured by OEP and by the smart textiles with the automatic method and the manual one, respectively.

The values of T_r_, T_i_, and T_e_ estimated by the smart textile and the OEP were compared for all the subjects by Bland-Altman analysis. An example, reported in Figure 4a,b, shows the analyses performed to compare T_r_ estimated by OEP with the one estimated by the smart textile using the automatic method and the manual one to select the peaks, respectively. The same analyses have been performed on the values of T_i_ (Figure 4c,d) and of T_e_ (Figure 4e,f).

**Figure 5:**
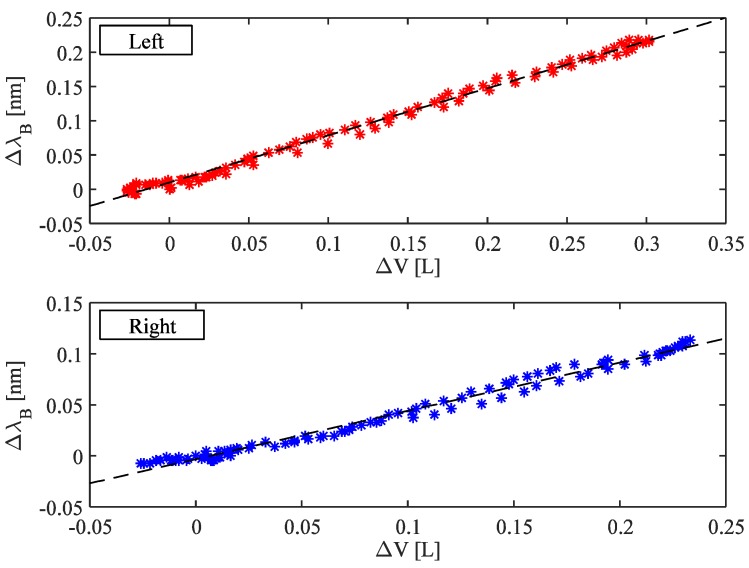
Correlation between the FBGs wavelength changes and UT volume considering both left and right side. The best fitting lines are also shown.

Figure 5 shows the wavelength changes of the left FBGs *vs.* the volume changes of UT_L_ obtained considering the same respiratory act (upper graph); the best fitting line is also shown. Figure 5 shows the same analysis performed on the right side (lower graph). The sensitivity of the system was calculated as the slope of the best fitting line.

Table 1 reports, for all the enrolled subjects, the mean value of the sensitivity, in both automatic and manual method to select the peaks. The table also represents the measurement percentage error of the system in tidal volume estimation calculated by Equation (2).

**Table 1:** Mean sensitivity of the textile for UT_L_ (K_L_) and UT_R_ (K_R_) and percentage error of the system for estimating UT_L_ (e_L_) and UT_R_ (e_R_) with both automatic and manual method of peak selection. These data are reported considering both the single subjects and the average value and standard deviation among all the subjects (last raw).

	K_L_ Automatic (nm∙L^−1^)	K_L_ Manual (nm∙L^−1^)	K_R_ Automatic (nm∙L^−1^)	K_R_ Manual (nm∙L^−1^)	%e_L_ Automatic (%)	%e_L_ Manual (%)	%e_R_ Automatic (%)	%e_R_ Manual (%)
Subject 1	0.66	0.71	0.43	0.48	13.05	11.42	12.73	10.35
Subject 2	0.68	0.69	0.31	0.35	5.82	5.8	10.39	5.82
Subject 3	0.88	0.87	1.19	1.17	7.23	10.13	8.85	8.93
Subject 4	0.65	0.66	0.61	0.64	5.05	5.48	6.05	6.5
All subjects (mean ± SD)	0.72 ± 0.11	0.73 ± 0.09	0.64 ± 0.39	0.66 ± 0.36	7.8 ± 3.6	8.2 ± 3.0	9.5 ± 2.8	7.9 ± 2.1

**Figure 6:**
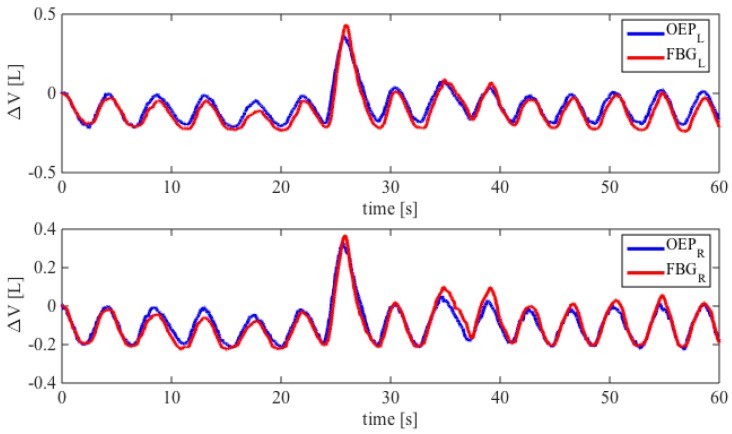
Comparison between the FBGs wavelength changes and UT volume considering both left and right side. The best fitting lines are also shown.

An example of the trends of volumes estimated by the proposed system and the OEP during quiet breathing is shown in Figure 6. Also a forced inspiration (7th acts) is shown. There are not notable differences between the data obtained by the two systems.

## 5. Discussion and Conclusions

This study present the design and preliminary assessment of a FBG-based smart textile for breathing monitoring. Its working principle, already assessed in the literature of this field, has been further investigated for the monitoring of T_r_, T_i_, T_e_, and semi-lateral UT volumes estimations.

The smart textile had a simple design and fabrication process, requiring one fiber housing two FBGs, a compliant polymeric material support to both cover and protect FBGs as well as to distribute the load caused by the UT movements.

FBGs and OEP traces have similar shape during all the breathing acquisitions. The smart textile shows good performances in terms of T_r_, T_i_, and T_e_ estimation. Bland-Altman plots show satisfactory accuracy for multiple subjects, with MOD lower than 0.045 s and 0.002 s in T_r_ estimation by adopting the automatic and the manual method, respectively. LOA analysis demonstrates that manual method (Figure 4b,d,f) has higher precision than automatic one in all subjects in the estimation of T_r_, T_i_ and T_e_. Moreover, both methods demonstrated a higher accuracy in the estimation of T_i_ and T_e_, for instance, in the subject showed in Figure 4, the MOD was 0.33 s and 0.35 s for T_i_ and T_e_, respectively. These encouraging results motivate our choice to use these two algorithms. More performant algorithms may be introduced in the future to extract other physiological parameters, as shown in [27].

Concerning the ability of the system to estimate the UT volumes, we found a high correlation coefficient (R^2^) between the FBGs wavelength changes and UT volume considering both left and right side (averaged-R^2^ was 0.87).

The analysis of textile sensitivity shows that this metrological property did not change significantly between manual and automatic method of peak selection. The sensitivity was always higher than 0.66 nm∙L^−1^ and 0.35 nm∙L^−1^ for the left and right side, respectively. This yields an averaged-volumetric error percentage of 8.3%, and comparable performances between the two methods of peak selection. The device performances are promising for real-time respiratory monitoring and for provide useful information regarding the detection of potential risks to health. Focusing on the inter-subject variability of sensitivity, it can be affected by the differences within chest wall circumferences and by the textile fabric creeping on the subject that can be produce slippage of sensors.

To the best of our knowledge, this is the first study in which the design of smart textile was led by a previous biomechanical analysis of chest wall movements during breathing, performed by using an OS. OSs are systems capable of very accurate measurements also for small deformations and displacement measurements [28]. The use of OS allows finding the best placement of FBGs to improve the sensitivity to strain of the entire smart textile. Usually, the positioning of the sensing element is not led by a quantitative analysis of the chest wall movements, but by a qualitative assessment. For instance, Witt and coauthors selected FBG sensors to monitor chest wall measurement and macrobending sensors for abdominal elongation. This proper choice was motivated by the fact that macrobending sensors are less sensitive than FBGs, hence they were used to monitor larger movements (abdominal) [1,10]. On the other hand they did not investigate the optimal positioning of the two sensors based on the different elongations experienced on the different parts of chest or abdominal wall. Moreover, the mentioned group analyzed in details the elongation experienced by the sensors, but they did not investigated the measurement accuracy of the smart textiles in terms of respiratory frequency and/or duration of respiratory phases. Regarding this topic, an interesting work was proposed by Dziuda *et al.*, which showed the accuracy of the proposed smart textiles in terms of respiratory rate (±1.2 respiratory per minute) [16]. A further interesting work was proposed by Silva and coauthors [29]. They developed a smart textile based on FBG technology able to monitor both cardiac and respiratory frequencies. The main innovation was the structure, consisting of a polymeric foil, in which the FBG was embedded.

In the present work, performances were analyzed by comparing the smart textile output and the OEP output during rhythmic breathing. In previous works [20], correlation with volume was investigated by using the spirometer outcome as reference. Spirometer measures inhaled and exhaled volumes of air from lungs while the proposed system measures overall volume changes of the torso. Hence, there are some inconsistencies between the two sets of results due to the fact that the sensing scheme measures volumetric changes in thorax which also includes physical muscular changes. Differently, OEP has the great advantage that it can measure breathing patterns and respiratory volumes in any condition, allowing partitioning of the complex shape of the chest wall into different compartments [30], taking account also deformities due to muscle recruitment. We focused on the UT volumes which allow us to monitor the chest wall deformities related to both alveolar pressure and rib cage muscles. Pulmonary rib cage (RCp), abdominal rib cage (RCa), and abdomen (AB) volume variations during quiet breathing reflect the different functional respiratory muscle groups (*i.e.*, rib cage muscles, diaphragm, abdominal muscles) activity and pressures (*i.e.*, pressure at the airway opening, alveolar pressure, pleural pressure, abdominal pressure, pressure at the body surface).

The encouraging results in monitoring UT foster further development to extend the functionality of the system in the monitoring of rib cage and abdominal compartments by adding others FBGs sensors on the textile. Moreover, the main limitation of the proposed system is related to the inter-subject variability. In fact, the pre-calibration of the system for each subject is not feasible in clinical settings due to the complexity of the calibration procedure, which involves the positioning of 89 markers on the subjects, then an off-line data analysis. In order to overcome this hurdle, the next step will be to face the variability of system sensitivity among subjects. The continuous measurement of thoracic and abdominal movements can be helpful regarding several clinical situations. For instance during anesthesia for MRI examination. In this procedure same drugs as anesthesia for any surgical procedure are used. Even if spontaneous respiration can be preserved most of the time, spontaneous respiration during anesthesia is constantly at risk of being impaired by anesthetic drugs or by upper airway obstruction. This system should also open new frontiers in the study of brain activity before and after respiratory rehabilitation by using the fMRI devices combined with the smart textile [31]. Moreover, the FBGs’ smart textile system can also provide an efficient solution for the sleep monitoring of infants or patients with sleep apnea and an alternative solution for the monitoring system which are based on resistive methods to assess the respiratory frequency during sport activities in athletes (e.g., elite cyclists). For these applications, where subjects’ motion can cause artifact to the system signal, *ad hoc* algorithms to reduce the motion noise should be applied and the performances of the system may be assessed during subject motion as reported in other applications [32].

Lastly, the design based on the analysis of chest wall displacements can pave the way for new strategy to optimize the sensitivity of smart textile for respiratory monitoring.
